# User Experience Estimation in Multi-Service Scenario of Cellular Network

**DOI:** 10.3390/s22010089

**Published:** 2021-12-23

**Authors:** Kaisa Zhang, Gang Chuai, Saidiwaerdi Maimaiti, Qian Liu

**Affiliations:** 1 Department of Information and Communication Engineering, Beijing University of Posts and Telecommunications, Beijing 100876, China; kaisa@bupt.edu.cn (K.Z.); saidi216@bupt.edu.cn (S.M.); 2 School of Communication and Information Engineering, Chongqing University of Posts and Telecommunications, Chongqing 400000, China; qianliu@cqupt.edu.cn

**Keywords:** users experience, naive Bayes, kernel density estimation, cellular network, network automation

## Abstract

The estimation of user experience in a wireless network has always been a research hotspot, especially for the realization of network automation. In order to solve the problem of user experience estimation in wireless networks, we propose a two-step optimization method for the selection of the kernel function and bandwidth in a naive Bayesian classifier based on kernel density estimation. This optimization method can effectively improve the accuracy of estimation. At present, research on user experience estimation in wireless networks does not include an in-depth analysis of the reasons for the decline of user experience. We established a scheme integrating user experience prediction and network fault diagnosis. Key performance indicator (KPI) data collected from an actual network were divided into five categories, which were used to estimate user experience. The results of these five estimates were counted through the voting mechanism, and the final estimation results could be obtained. At the same time, this voting mechanism can also feed back to us which KPIs lead to the reduction of user experience. In addition, this paper also puts forward the evaluation standard of the multi-service perception capability of cell-level wireless networks. We summarize the user experience estimation for three main services in a cell to obtain a cell-level user experience evaluation. The results showed that the proposed method can accurately estimate user experience and diagnosis abnormal values in a timely manner. This method can improve the efficiency of network management.

## 1. Introduction

5G has brought great changes to life, and many new application scenarios have appeared, such as auto driving, AR, VR, and so on. At the same time, cellular traffic has increased greatly [1]. Ultra-dense deployment of 5G makes the cost of network construction increase, and the difficulty of network management has also increased. Users have higher requirements for real-time experience than before. The balance between resource consumption and users’ low-latency and highly reliable experience is a huge challenge for operators. Since operators need to consider both costs and users’ real-time experience, to solve this problem, quality of service (QoS) and quantity of experience (QoE) are topics that cannot be separated. Research on network automation based on the estimation of the QoE by QoS is becoming more and more popular.

The QoS is a performance indicator of every network, and it can be truly measured and used in network services’ improvement. It is worth noting that for any wireless network, whether 3G, 4G, or 5G, the measurement indicators of network performance are basically the same. The QoS has always been the focus of research and the basis of network management. However, QoS parameters only reflect the network performance, which can not directly reflect the user satisfaction [2]. The specific indicators of the QoS are the KPIs, which are often the data collected from the base station in a cellular network. The indicator of the QoE is different from the QoS, which is composed of subjective and objective parts. The subjective part often varies from person to person; the judgment standard is usually the mean opinion score (MOS). The objective part is the key quality indicators (KQIs). The objective part is the focus of our research.

The goal of operators is to provide users a satisfactory level of the QoE during service delivery. There is a strong relationship between the QoS and QoE. Usually, QoS parameters have a great impact on the QoE. The standardized LTE network focuses on defining network performance from the perspective of the QoS and does not define the relationship between two entities. A complete quality analysis needs this relationship in order to maximize the QoE during service delivery. The expected level of the QoE can be estimated through the QoS/QoE model according to the QoS measurement results [3]. At present, most of the research on the QoE focuses on the subjective index MOS. Although the mapping of the MOS-QoE [4] can well judge the perception of users, it is obviously not realistic to obtain the subjective opinions for each user in the actual network optimization with tens of millions users.

**Figure 1:**
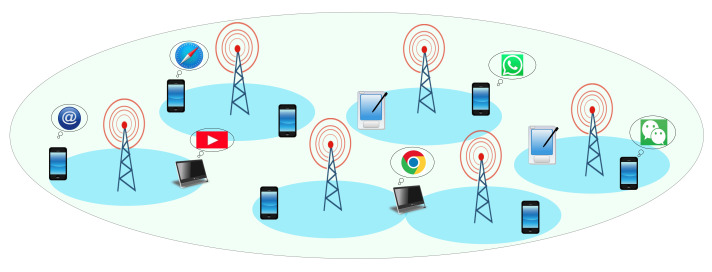
Main service types of cellular network users.

For cellular operators, considering the ultra-dense deployment of 5G and the challenges brought by the low latency requirement, it is necessary to provide the guarantee of user experience for key services. At the same time, energy consumption should also be considered. Therefore, the purpose of this paper is to estimate user experience for multiple services through KPIs and combine experience estimation with fault diagnosis. For users, there are three widely used main types of service. As shown in Figure 1, these three types of applications are the most frequently used in daily life. Instant messaging includes WeChat, WhatsApp, and other applications. Video service is provided by YouTube, TikTok, etc. Web page browsing is also a common type of application, including news, email, etc. We chose these three main service types for user experience estimation and fault diagnosis. User experience for these three types of services are summarized as cell-level wireless network perception capabilities, which provide direct assistance for network management. At the same time, faults can be diagnosed in time when the user experience is estimated to decline.

A method for user experience estimation and diagnosis for cellular network is proposed in this paper. The main contributions are as follows:

First, due to the complexity of KPI data in wireless networks, the probability density distribution of various data is different. Therefore, in order to improve the estimation accuracy, an adaptive selection method for the kernel and bandwidth based on the naive Bayesian classifier is proposed. Through this mechanism, an accurate association between KPIs and KQIs is realized.

Second, in this paper, KPIs for each type were used to obtain the corresponding estimates of multiple KQIs, and the final estimation result of the KQIs can be obtained through a voting mechanism. When the estimated value for the KQIs is abnormal, we can analyze the causes of network failure according to the votes of different types of KPIs, that is, when the user experience is predicted to degrade, the cause of the fault can be found in time. At the same time, the accuracy of estimation is also guaranteed.

Third, through the user experience estimation of three main services, we propose a method to evaluate the perception capability of cell-level wireless networks. As far as the authors know, this is the first time cell-level multi-service perception estimation has been proposed. This method can deal with user experience optimization of different services in cell-level cellular networks.

## 2. Related Works

In recent years, the research of wireless network performance prediction has also received extensive attention, most of which is based on KPIs, and KQIs are the reaction of the actual user experience. As mentioned above, with the continuous acceleration of technological progress, more users access services such as video, audio, and other multimedia applications on cellular networks [5]. Mobile network operators need to continuously respond according to user needs. User-centric network management can be seen as the key work of network providers [6]. The concept of the QoE is gaining more attention. K. Bouraqia et al. [7] introduced the importance of the QoE in wireless networks. The QoE is used to replace the QoS to enhance user feedback on specific services. K. Bouraqia et al. [7] proposed that the QoE can be divided into two parts: objective (network objective indicators) and subjective [8] (personal experience obtained from expectations, emotional states, feelings, preferences, etc.). In other words, it is an assessment of an individual’s experience when interacting with service [9]. In order to consider user satisfaction in the context of real-time video streaming applications, the QoS is no longer sufficient to assess quality. However, from the perspective of time series analysis, KQIs for different services are often unpredictable, because their values are always percentage numbers, such as drop call rate, video playback success rate, etc. Our research focuses on the KQI index, which is the objective part of the QoE. A KQI estimation method is very helpful for operators to better guarantee users’ real-time experience. At the same time, it also brings convenience to the optimization of the network. Combined with wireless network performance prediction, KQI estimation can enhance the practical significance of KPI prediction.

The focus of network management and optimization has gradually shifted to the user experience. User perception, commonly known as the quality of experience (QoE) index, has become one of the most important topics for network service providers (NSPs) and content service providers (CSPs). As mentioned above, user experience can be divided into subjective and objective parts. In [10], the author proposed a new QoE estimation method based on subjective evaluation machine learning (ML) in a laboratory environment. The author combined QoS parameters and emotional computing (facial expression) to estimate the MOS of video service. The evaluation using the collected subjective datasets showed that the combination of the QoS and affective computing can provide better prediction performance. In [11], the authors also mentioned that the QoE is a widely concerned research topic, which provides a method to analyze the general acceptability of applications or services perceived by users. This perception will be affected by user expectations and their environment. The QoE can be estimated through subjective indicators obtained from questionnaires and/or surveys and/or objective indicators automatically calculated from devices. Their work presented the results of a 17 d unsupervised field study in Rio Gallegos, Argentina. In [12], the author put forward a machine-learning solution, which is based on the network-related system influence factors (SIFs) as the input data to perform multidimensional prediction for the QoE. The proposed solution was verified by the experimental research based on an LTE video stream simulation. The final result combined the objective evaluation of KQIs and the subjective evaluation of the MOS. In [13], the authors showed how the QoE is affected by the channel bandwidth, task content, user personality, and gender through Bayesian statistical analysis. They indicated that quantitative evaluation of the QoE in multimedia communication is a challenging problem. In [14], through the statistical analysis of network video stream data, authors used the ITU-T p.1201 1 model to find a correlation between the QoE and video service QoS parameters. From the perspective of user experience, the QoS recommendation index of each MOS value interval could be given, and the connection between the QoS and QoE was established.

Most research uses the MOS in QoE analysis. For wireless network operators, it is difficult to grasp the subjective recognition of each person of the experience. Therefore, some research focuses on the objective part of user experience and emphasizes the practicability of the research. The purpose of [15] was to use a data-driven architecture for personalized QoE management in a 5G wireless network, which consumes less resources. In this work, a machine-learning algorithm was used to predict the QoE through QoS parameters. They created a 5G simulation environment reflecting the current mobile traffic pattern to obtain datasets for training. The objective tool of video QoE evaluation was used to collect QoE data for training the prediction model. Support vector machine, random forest, the gradient lifting tree, and a neural network were selected as the machine-learning algorithms for experience prediction, and the results showed that they achieved high accuracy. In [16], the author also thought that cellular operators have changed their focus from the QoS to the QoE. The author mentioned that although more advanced schemes have been designed to balance the QoE among cells, these methods cannot guarantee the improvement of the overall system QoE. In this work, they proposed a new self-tuning algorithm based on the QoE for classical mobile load balancing schemes. References [17,18] thought that although the quality of service can be used to improve the network quality, it does not represent user satisfaction. Therefore, it is very important to use QoE and QoS parameters to evaluate and improve network quality. In the research of [17], they put forward the modeling of the QoE based on QoS parameters and used the artificial neural network (ANN) method to evaluate and synthesize the QoE in the actual environment with the help of a road test. Reference [18] introduced the QoE into the decision-making mechanism of vertical handoff and proposed a QoE estimation based on a stochastic neural network to determine the correlation between the QoE and QoS in heterogeneous networks. In [19], the author considered specific key performance indicators (KPIs) and suggested using neural networks to provide automatic classification between these KPIs and QoE to ensure the consistency of user experience. N. Ahmad [20] pointed out that most existing studies on QoS to QoE mapping ignore any correlation among QoS indicators. They analyzed the real-time cellular QoS index and observed significant correlations between several QoS parameters. Different statistical methods (such as maximum likelihood estimation and cumulative distribution function) were used for different parameters. Customized simulators were used to observe the impact of related delay loss indicators on video streaming end-user QoE.

In specific association research, the association between KPIs and KQIs is often regarded as the problem of classification; the results of classification are the different states of the KQIs. Reference [21] proposed a quantitative association rule mining (QARM) method based on machine learning, which is called SWP-RF. It consists of sliding window partition (SWP) and random forest (RF). Specifically, SWP is used to discretize continuous attributes into Boolean values to mine association rules. The warning interval and warning point are obtained by SWP. Next, the author used the importance of RF features to measure the association between KPIs and KQIs. RF can be used to obtain the priority of the correlation strength between all KPIs and each KQI. Finally, they selected warning points and correlation strength priority as the optimal output solution. Actual data from telecom operators were used in experiments, and the results verified the feasibility and accuracy of proposed method. It was mentioned in [22] that users will not directly perceive the impact of QoS, but evaluate user experience according to a variety of QoSs in the whole system. An ML algorithm based on support vector machine (SVM) was selected to complete the correlation between the QoE and QoS. The authors in [23] proposed that in QoE management, networks and service providers usually rely on a model that maps system QoS conditions (such as system response time, packet loss, etc.) to the end-user QoE value. In [23], a mapping function from the QoS to MOS was studied, and the authors derived corresponding QoE scores according to this user rating distribution. Y. Ben et al. [24] proposed a novel online QoE estimation model, which used incremental multiclass SVM (multiclass-iSVM) to classify users’ perception of video streaming services. The proposed online QoE model studies the effectiveness of incremental learning to process large-scale dynamic data and improve the prediction accuracy of the QoE. In [25], support vector machine (SVM) was also used to estimate the QoE. This method achieves a trade-off between the learning ability and computational complexity of QoE estimation. R. Elwerghemmi et al. [26] pointed out that the machine-learning model provides a solution for obtaining the complex relationship between various influencing factors and the QoE. The proposed method based on SVM was suitable for processing non-stationary data. The experimental results proved that the proposed model has good results in terms of classification accuracy and computational complexity.

In this paper, we also estimated KQIs, which are the objective part of the QoE. The relationship between KPIs and KQIs was also transformed into a classification problem. Considering the data complexity of KPIs in wireless network, we did not use the discrete method, but based on the combination of kernel density estimation (KDE) and naive Bayes. KDE is also widely used in parameter estimation. A. Eckert-Gallup et al. [27] suggested using bivariate kernel density estimation (KDE) with adaptive bandwidth selection to generate an effective joint probability distribution. Y. Shitara et al. [28] pointed out that the QoE is often affected by many factors. Therefore, collecting QoS data from users who use mobile applications has become a promising solution for the QoE, called crowdsourcing data. However, crowdsourced data are susceptible to perception errors and low accuracy. In their research, a kernel density estimator was used to derive a continuous density function from discrete distributed sample data. Simulation experiments verified the effectiveness of the proposed method. T. Kinoshita et al. [29] proposed a data-driven control scheme by using kernel density estimation. Kernel density estimation can calculate the similarity between the query data and the database. According to the proposed scheme, the controller parameters were calculated based on the anomalies obtained through the above-mentioned similarity. Reference [30] optimized the KDE algorithm by minimizing the mean integrated squared error (MISE) and selected the most suitable bandwidth. The purpose of [31] was to compare the optimization of grid search parameters with the genetic algorithm to determine the bandwidth parameters of the naive Bayesian kernel density estimation algorithm. The parameters used to compare the two optimization methods included precision, area under curve (AUC) and calculation time. Reference [32] proposed a semi-naive Bayesian method based on KDE. The author also mentioned that the method of KDE was used instead of data discretization. The correlation of probability density was defined to find the best probability density distribution. Reference [33] proposed a method, the Naive Bayes classifier for continuous variables using novel method (NBC4D), which mainly combines KDE with naive Bayes. The author chose a variety of probability density distribution functions, such as the Gauss index, Rayleigh distribution, etc. The optimal kernel function was found to be used in the classifier by comparing the relationship between the distribution function of probability density and the distribution of original data by the variance and mean.

Considering the complexity of KPI data in wireless networks, we also used KDE to estimate user experience. In this way, accuracy is guaranteed, and the discretization operation is not needed in the prepossessing part. We propose an adaptive bandwidth selection method for KDE. It can be seen that the estimation of user experience is mainly concentrated in a certain service [10,12,13,15]. In this paper, we propose a cell-level wireless network perception capability, and we chose three main service types in the analysis of user experience. These three types of services are web browsing, instant messaging, and video services. At the same time, it can be seen from the current research that most of the user experience estimation does not consider the combination of fault diagnosis and estimation. In this paper, we introduced a voting mechanism to bring fault diagnosis into the research of user experience. In this way, user experience can be monitored in real time. When the degradation of user experience is estimated, the fault causes can be found out in time and network optimization can be carried out. For the estimation algorithm based on naive Bayes, KDE with an adaptive bandwidth was used to improve the prediction accuracy and further improve the efficiency of network management and optimization.

## 3. Materials and Methods

### 3.1. Data Description

The data used in simulation came from a real cellular network, which were provided by an operator in China. We used the data of a base station over 104 days (15 July 2019–27 October 2019) to correlate the KPIs with the KQIs. The data were collected every hour, and the statistics was the average value within one hour. Therefore, for each kind of KPI/KQI, a total of 2496 pieces of data was collected. There were 1500 pieces of data used for training, and the remaining 996 pieces of data comprised the test set.

KPI data were divided into five categories, as shown in Table 1. Accessibility KPIs include the successful establishment of the number of RRC connections and E-RAB. RRC_establish reflects the acceptance capability of the UE (user) in a eNB (eNodeB) or a cell. The successful establishment of RRC connection means that the UE has established a signal connection with the network, which is the basis for other services. E-RAB_establish means that eNB successfully allocates the connection to the UE, which reflects the ability of eNodeB or the cell to accept services. E-RAB establishment is used to transmit voice, data, and multimedia services between the UE and core network (CN). The establishment of E-RAB is initiated by the CN. After E-RAB is successfully established, a basic service is established, and the UE enters the service process. Mobility KPIs are the statistics of handover KPIs in a cellular network. Handover can be divided into the same frequency and different frequency according to the carrier frequency configuration. The handover capability directly affects the satisfaction of users with the network services. The success rate of intra-eNB handover reflects the continuity of users’ service in movement. It is related to the handover processing capacity and network planning, and users can directly feel it. Network quality includes Uplink_MAC_block_error and Downlink_MAC_block_error. They reflect the system MAC layer load and represent network load and system processing capacity to a certain extent, wherein downlink refers to eNB to the UE and uplink refers to the UE to eNB. Capacity includes the average utilization of the PUSCH PRB and PDSCH PRB, in which, PRB is the physical resource block. They represent the PRB usage assigned to PUSCH and PDSCH, respectively. These indicators reflect the utilization of the wireless resources of the system and provide a basis for system algorithm optimization. It is worth noting that the fifth type of data was set as a potential factor, because the number of users and traffic volume are constantly changing over the long term. This is a potential variable for network construction. The KQI data are shown in Table 2. We selected one KQI for each service type.

**Figure 2:**
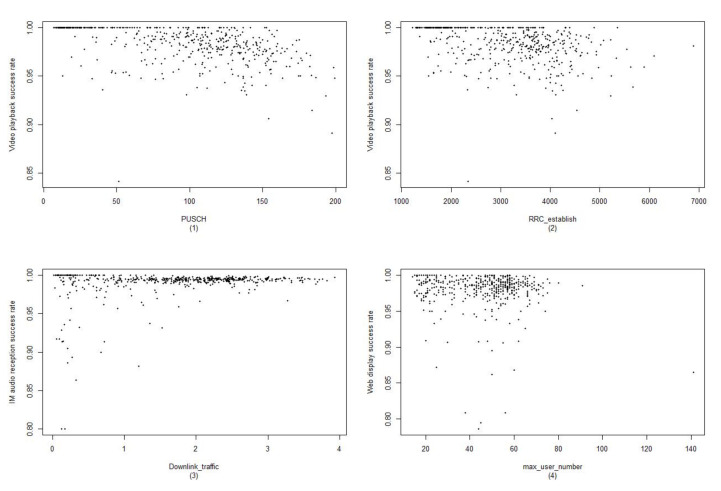
In Figure 2: (1) shows the corresponding relationship between the video playback success rate (KQI) and the PUSCH utilization rate (KPI) in the cell; (2) shows the corresponding relationship between the video playback success rate and the number of RRCs established in the cell; (3) shows the corresponding relationship between the IM audio receiving success rate and the downlink traffic in the cell; (4) is the corresponding relationship between the web display success rate and the max number of the cell.

As can be seen from Figure 2, the change of the KPIs and KQIs are not linear, and the corresponding relationship is irregular. Therefore, if we discretize the KPI data, we cannot obtain the relationship well, and we will lose some information in the process of model learning. Therefore, discretization was not considered in the data processing. The probability density distribution was used in the classifier. As mentioned above, we combined the estimation of user experience and fault diagnosis. Therefore, each kind of KPI data will have the corresponding fault type. When a certain kind of KPI is associated with the deterioration of the KQI, the possible cause of the failure can be found directly. The relationship between the KPI and corresponding fault causes [34] is given in Table 3.

### 3.2. Methods

The purpose of the proposed method is to estimate KQIs for different services. At the same time, fault diagnosis and optimization suggestions are given. As mentioned above, most of the research on cellular KQI estimation is based on discrete KPI data. Due to the complexity of KPI data, discretization will bring much inaccuracy. Some commonly used association methods, such as a priori [35], SVM [24,25], random forest [36], and so on, have a high complexity, and these methods cannot obtain real-time fault cause feedback.

We did not discretize KPI data, but KQI data were discretized by converting them into Boolean values (abnormal/normal) through the threshold value. The threshold value is adjustable, which can be changed according to the requirements of network optimization personnel. In the evaluation, the threshold for the KQIs of their service types (web response success rate, IM receiving success rate, and video playback success rate) was set to 95%. In this way, the estimation of user experience was transformed into a binary classification problem. When the KQI was lower than 95%, it was judged as abnormal, and when the KQI was greater than or equal to 95%, it was judged as normal. In this way, the estimation of user experience was transformed into a binary classification problem. We used the method based on naive Bayes. Through the combination of KDE and the adaptive bandwidth selection algorithm, the optimal kernel function was found. When choosing the optimal kernel function, we used the $R^{2}$ goodness of fit as the standard. Finally, the kernel function was applied to the classifier.

**Figure 3:**
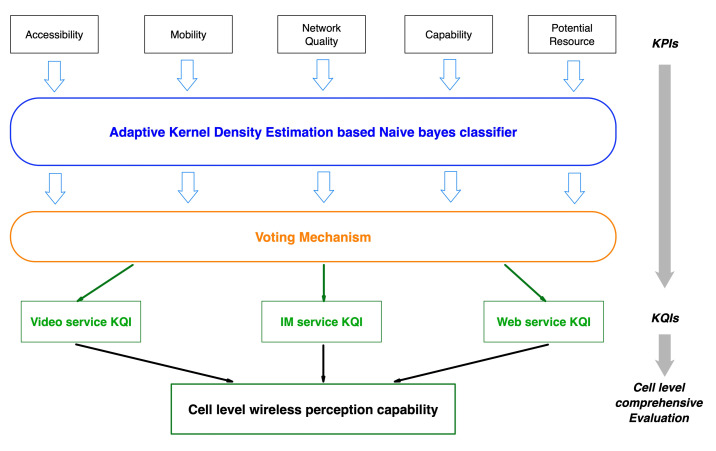
User experience estimation.

KPI data of different classes as shown in Table 1 were put into different classifiers for processing. Five KQI estimation results could be obtained, and user experience estimation results for each service type could be obtained through the voting mechanism. The user experience for three service types was summarized into a cell-level evaluation. Figure 3 shows these processes.

Because of the complexity of KPI data, the probability density distribution of each type of data is different. Therefore, different Bayesian classifiers were used to process these data, and different bandwidth and kernel functions were considered. For each kind of KPI data, the estimated value of KQI was obtained respectively, and then, the final KQI estimation was obtained by a voting mechanism. This can improve the accuracy of prediction, and more importantly, when the KQI degradation is estimated, we can analyze the causes of network failure according to the voting of different types of KPIs. The table below shows the corresponding relationship between different types of KPI degradation and failure causes. According to the KQI of each service, the overall wireless network service perception ability of the cell is judged.

(1) $$\begin{array}{c} S={\left[{\left[{\left[s_{m,t}\right]}_{t=1}^{T}\right]}_{m=1}^{M}\right]}_{g=1}^{G} \end{array}$$

In Equation (1), *S* is the KPI dataset, *t* is time, and *m* is the KPI type, where maximum value of *m* is 5, and five KPI types (access, handover, network quality, capacity, potential resources) were used in the evaluation. *g* is the service type, and there are three service types (instant messaging, video, and web browsing) in total. For KQI data, Boolean conversion was carried out according to the threshold value to construct set *C*, as shown in function (2), where 0 represents abnormal and 1 represents normal. The threshold value can be set according to the actual network management and optimization requirements.

(2) $$\begin{array}{c} C=\left\{0,1\right\} \end{array}$$

In this way, the estimation of the KQI can be transformed into a binary classification problem. According to the idea of naive Bayes, our goal was to classify the KQI according to the conditional probability.

#### 3.2.1. Adaptive Kernel Density Estimation

Solving the distribution density function of random variables from a given sample set is one of the basic problems in probability statistics. Methods to solve this problem include parametric estimation and nonparametric estimation. Parameter estimation can be divided into regression analysis and discriminant analysis. In parametric regression analysis, people assume that the data distribution conforms to a certain property, such as a linear, reducible linear, or exponential property, and then, a specific solution can be obtained in the objective function sets. Experience and theory show that there is a gap between the basic assumption of the parametric model and the actual model. These methods cannot always achieve satisfactory results.

Due to the above defects, a non-parametric estimation method was proposed [37], namely kernel density estimation. Kernel density estimation does not use the prior knowledge about the data distribution and does not attach any assumptions to the data distribution; it is a method that can capture the characteristics of the data distribution from the data sample itself. Therefore, it is highly valued in the field of statistical theory and application [38].

(3) $$\begin{array}{c} f_{h}\left(x\right)=\frac{1}{nh}K_{n}\left(x-x_{i}\right)=\frac{1}{nh}\sum_{i=1}^{n}K\left(\frac{x-x_{i}}{h}\right) \end{array}$$

In Equation (3), K(.) is a kernel function. h>0 is a smoothing parameter called the bandwidth. $x_{1},x_{2},x_{3}\ldots x_{n}$ represents n sample points. In the kernel density estimation part, we selected the kernel function and the optimal bandwidth, respectively. Considering the different data distribution for each KPI, four kernel functions were used for simulation, and they are Gaussian, Epanechnikov, cosine, and rectangular.

**Figure 4:**
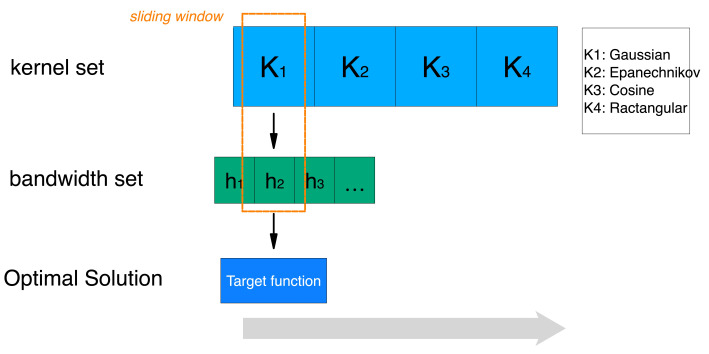
Two-step optimization for kernel density estimation.

In order to select the optimal kernel function and bandwidth, we propose a two-step optimization scheme. As shown in Figure 4, the kernel function and bandwidth were successively selected through a sliding window. Specifically, we first selected one of four kernel functions, then found the optimal bandwidth based on the known kernel function. When the optimal bandwidth of each kernel function was selected, we chose the optimal kernel density estimation scheme from these four cases and used it in the classifier. The specific algorithm is given by Algorithm 1.
**Algorithm 1** Two-step optimization in kernel density estimation.**Require:** *K*: Kernel function set; *S*: KPI set;**Ensure:** optimal $K^{*}$; optimal $h^{*}$ 1: Initial $K_{i}$ and *h*; 2: Calculation of average value and standard deviation for each attribute in *S*; 3: **repeat** 4:  Kernel function selection; 5:  **repeat** 6:   Bandwidth calculation; 7:  **until** Based on current kernel function, the optimal bandwidth $h^{*}$ is found 8: **until** The optimal kernel function $K^{*}$ and optimal bandwidth $h^{*}$ are obtained

Once the kernel function has been determined as Equation (3), when selecting the bandwidth, the optimal bandwidth for the current kernel function can be found through the MISE.

(4) $$\begin{array}{c} MISE_{h}=E\int {\left(f_{h}\left(x\right)-f\left(x\right)\right)}^{2}dx \end{array}$$

The MISE in Formula (4) can be decomposed into three parts as:

(5) $$\begin{array}{c} MISE_{h}=\int Ef_{h}^{2}\left(x\right)dx-2\int f\left(x\right)Ef_{h}\left(x\right)dx+\int f^{2}\left(x\right)dx \end{array}$$

The third component in Equation (3) was removed because it does not depend on the kernel, and the cost function is defined as:

(6) $$\begin{array}{c} {\hat{C}}_{h}=MISE-\int f^{2}\left(x\right)dx \end{array}$$

which also can be expressed as:

(7) $$\begin{array}{c} {\hat{C}}_{h}=\int Ef_{h}^{2}\left(x\right)dx-2\int f\left(x\right)Ef_{h}\left(x\right)dx \end{array}$$

The minimizer of ${\hat{C}}_{h}$ is an estimate of the bandwidth in Algorithm 1, which is denoted by $h^{*}$.

When comparing different kernel functions with the corresponding bandwidth, the $R^{2}$ goodness of fit was used as the comparison standard. The goodness of fit refers to the fitting degree between the regression model and the observed value. Statistical measurement of the goodness of fit was the determinate coefficient $R^{2}$. The maximum value of $R^{2}$ is 1. The closer to 1, the better the fitting degree of the regression to the observed value that can be obtained; on the contrary, the smaller $R^{2}$ is, the worse the fitting degree of the regression to the observed value is.

*y* is the value to be fitted; the mean value is $\bar{y}$, and the fitting value is $\hat{y}$. The sum of squares total (SST), sum of squares regression (SSR), and sum of squares error (SSE) are expressed as follows:

(8) $$\begin{array}{c} SST=\sum_{i=1}^{N}{\left(y_{i}-\bar{y}\right)}^{2} \end{array}$$

(9) $$\begin{array}{c} SSR=\sum_{i=1}^{N}{\left(\hat{y_{i}}-\bar{y}\right)}^{2} \end{array}$$

(10) $$\begin{array}{c} SSE=\sum_{i=1}^{N}{\left(y_{i}-\hat{y_{i}}\right)}^{2} \end{array}$$

According to SST=SSR+SSE, $R^{2}$ can be obtained by Equation (11).

(11) $$\begin{array}{c} R^{2}=\frac{SSR}{SST}=\frac{\sum_{i=1}^{N}{\left(\hat{y_{i}}-\bar{y}\right)}^{2}}{\sum_{i=1}^{N}{\left(y_{i}-\bar{y}\right)}^{2}}=1-\frac{SSE}{SST} \end{array}$$

**Figure 5:**
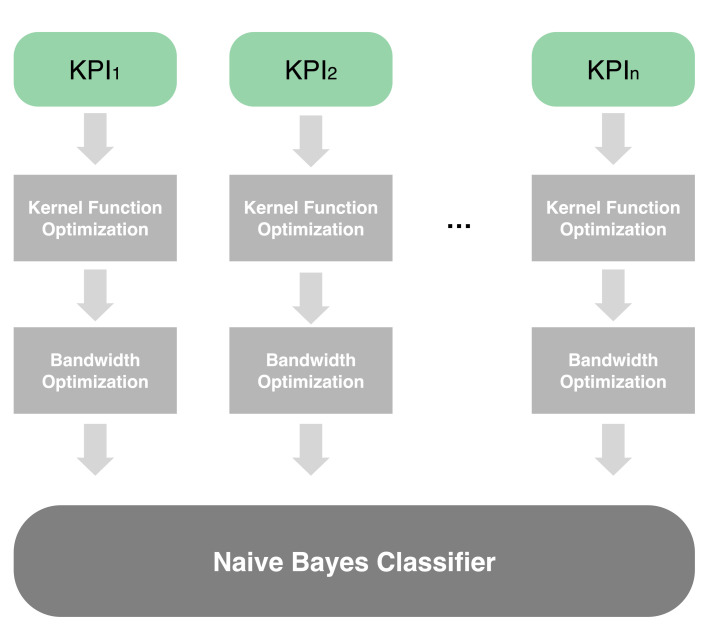
Two-step kernel density estimation.

We adopted a two-step KDE method to select the kernel function and bandwidth according to the determination coefficient. As shown in Figure 5, we selected the most suitable kernel function and bandwidth for each group of KPI data. The final probability density distribution function was used in the naive Bayes classifier.

The essence of kernel probability density estimation is to regard each datum and bandwidth as the parameters of the kernel function. After multiple kernel functions have been obtained, the kernel density estimation was formed through linear superposition, then the kernel density probability density function could be obtained after normalization. We used this method in the probability calculation, and the result can be used in the naive Bayesian classifier. This can improve the estimation accuracy and avoid the error caused by the discretization of KPI data. In kernel density estimation, the bandwidth determines the smoothness, and the kernel function determines the shape of the distribution.

#### 3.2.2. Naive Bayes Classifier

The naive Bayes classifier is a series of simple “probability classifiers” based on the application of the Bayesian theorem. It is also a supervised machine-learning technology for classification and characterized by being fast, accurate, simple, and easy to operate [39]. Each input variable in naive Bayes is assumed independent.

We divided the KQIs into two categories: normal and abnormal, represented as 1 and 0 according to the threshold value. The conditional probability can be obtained as $P\left(S|C\right)$. For the KQI estimation, the classification problem can be expressed as:

(12) $$\begin{array}{c} P\left(C=0|S\right)=P\left(C=0\right)\prod_{i=1}^{n}P_{i}\left(s_{i}|C=0\right) \end{array}$$

(13) $$\begin{array}{c} P\left(C=1|S\right)=P\left(C=1\right)\prod_{i=1}^{n}P_{i}\left(s_{i}|C=1\right) \end{array}$$

The judgment of the result can be expressed as:

(14) $$\begin{array}{c} log\left(\frac{p\left(C=0|S\right)}{p\left(C=1|S\right)}\right)=log\frac{p\left(C=0\right)}{p\left(C=1\;\right)}+\sum_{i=1}^{n}log\frac{P_{i}\left(s_{i}|C=0\right)}{P_{i}\left(s_{i}|C=1\right)} \end{array}$$

We define the threshold Θ, if $log\left(\frac{p\left(C=0|S\right)}{p\left(C=1|S\right)}\right)⩾\Theta$, and the KQI is considered abnormal.

For continuous data such as the KPIs in this paper, we used the following probability density function for naive Bayes.

(15) $$\begin{array}{c} f\left(s\right)=\frac{1}{nh}\sum_{i=1}^{n}K\left(\frac{s-s_{i}}{h}\right) \end{array}$$

where *K* and *h* are provided by the last step. s∈S and $s_{i}$ are the training points. For a certain KPI, the conditional probability can be expressed as:

(16) $$\begin{array}{c} P\left(s|C=c_{k}\right)=\frac{1}{nh}\sum_{i=1}^{n}K\left(\frac{s-s_{i}}{h}\right)\left[C=c_{k}\right] \end{array}$$

where $c_{k}\in \left[0,1\right]$ is the classification set. After kernel function optimization and bandwidth optimization, the probability density distribution function corresponding to each group of KPIs is used in the classifier.

#### 3.2.3. Voting Mechanism

**Figure 6:**
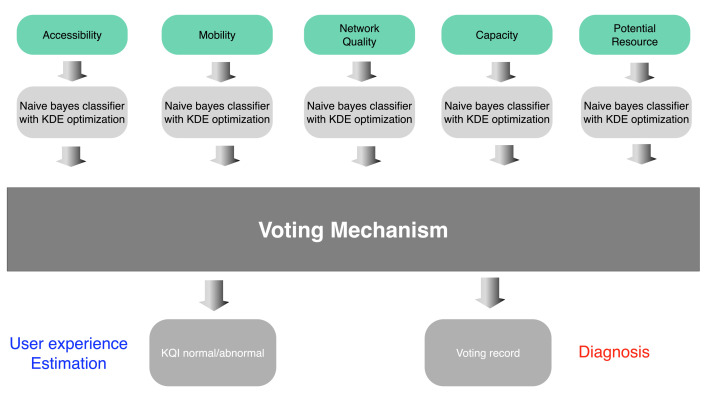
Voting mechanism.

Different from other methods using the Bayesian classifier for estimation, we classified KPI data. For each class of KPIs, the KQI can be estimated respectively. The estimated results of each category are summarized by the voting mechanism. In all results, if more than two abnormal KQI results were estimated, we considered that the KQI value was abnormal. As shown in Figure 6, this is the judgment process of voting mechanism. It should be noted that the voting information will be collected every time. We can judge the main reason for the decline of user experience according to Table 3 in the cell through the statistics of multiple voting.

As mentioned above, we divided the KPIs into five categories. One of three services’ KQI was selected, which means the user experience of one service type would be estimated. At the same time, five kinds of KPIs were used to estimate the KQI one by one. It can be seen from function (1) that when we estimate the first service type KQI through the first type of KPI data, we set m=1 and g=1, and the KPI data can be expressed as $S_{m_{1},g_{1}}$, while the correlation result can be expressed as $C_{m_{1},g_{1}}$. Each type of KPI was associated with the first KQI one by one. The summary result can be expressed as:

(17) $$\begin{array}{c} C_{m_{1},g_{1}}=\sum_{m=1}^{6}C_{m_{i},g_{1}} \end{array}$$

When summarizing the results, we chose the voting mechanism. When the proportion of abnormal KQIs estimated by the KPIs accounted for more than two, the current service KQI was determined as abnormal. This judgment method is expressed as Equation (18). Finally, the KQI results estimated by each type of KPI were recorded. The statistical result for every vote can be used for fault diagnosis.

(18) $$\begin{array}{c} \left\{\begin{array}{cc} C_{m,g}>2 & Normal\\ C_{m,g}\leq 2 & Abnormal \end{array}\right. \end{array}$$

#### 3.2.4. Service Perception Capability in Cell-Level Wireless Networks

The perception capability of wireless networks is a comprehensive reflection of users’ experience of multiple services in a cell-level wireless network. In this paper, we considered three main services, and the user experience of these three main services was used to evaluate the perception capability of wireless networks. In actual network optimization, different service types can be assigned different weights to obtain the overall user experience according to the requirements of network management. Through the Bayesian classifier and the voting mechanism, the user experience of each kind of service in the cell can be obtained. We summarize the KQI of three services (instant messaging, video, and web browsing). As shown in Table 4, these four levels depend on the proportion of abnormal KQIs in three services. For example, if user experience estimates of two from three services were abnormal, the cell-level user experience was considered poor. When the user experience of three services was estimated to be normal, the cell-level user experience was evaluated as excellent. So far, the cell level wireless perception capability evaluation has been completed.

## 4. Results

### 4.1. Evaluation of the Adaptive KDE Naive Bayes Classifier

The KPI types are given in Table 1, and the KQIs of three services are estimated, respectively. The corresponding KQIs of the three services are video playback success rate, instant messaging audio transmission success rate, and web response success rate. In the verification, naive Bayesian classifier based on the Gaussian kernel function (GNB), and K-nearest neighbor (KNN) were used as the comparison algorithms. AKNB is our proposed algorithm, which means adaptive kernel density estimation based naive Bayes. For the KNN, we set the clustering number *K* to two. The ratio of the training set to the test set was set to 1.5:1. The verification results of the three services’ KQIs are shown in Table 5. Through the results shown in these three tables, it can be seen that compared with the other two common methods, naive Bayes based on KDE with two-step optimization had better accuracy for the association between KPIs and KQIs.

**Figure 7:**
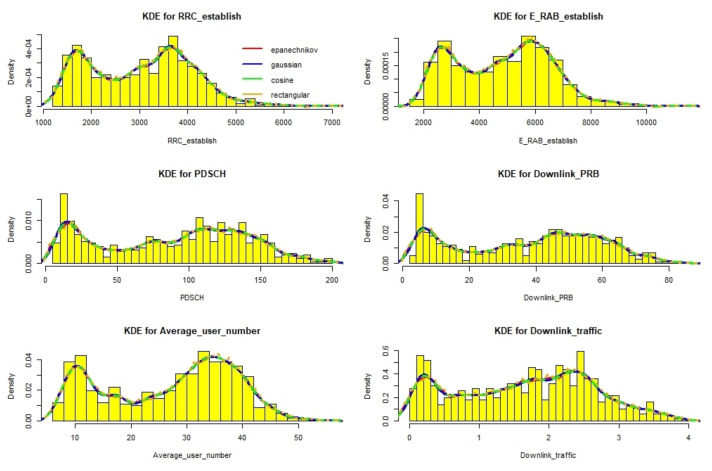
Kernel density estimation results for different KPI data.

**Figure 8:**
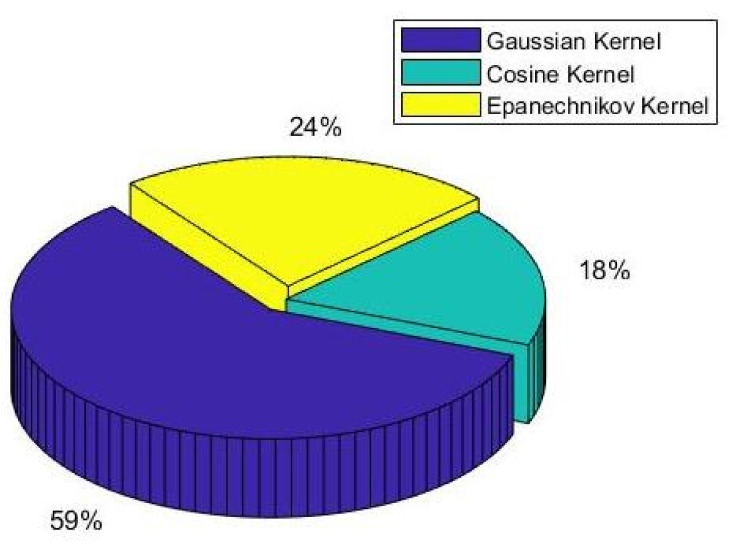
Proportion of each kernel function.

At the same time, we explored the selection of the kernel function in the two-step optimization. Figure 7 shows the kernel density estimation results of the four kernel functions for different KPI data. From Figure 8, it can be seen that the Gaussian kernel function was the most selected one when performing adaptive kernel density estimation for all KPI data.

### 4.2. Evaluation of the Combination of Classifiers with the Voting Mechanism

In order to combine user experience estimation and network fault diagnosis, a voting mechanism was introduced into our method. As mentioned above, our estimation of cell-level KQI was obtained from the estimation results of five types of KPIs, which were counted by the voting mechanism. The fault diagnosis was based on the vote (normal or abnormal) of each type of KPI.

Taking the video service as an example, we verified the estimation accuracy of this voting mechanism. The KNN and SVM based on multiple kernel functions were used for the comparison. SVM is an advanced algorithm commonly used in user experience estimation and fault diagnosis. For the proposed method, we first obtained the estimated value of each kind of KPI for the video service KQIs according to the adaptive KDE naive Bayes based on two-step optimization, and then, we summarized the five results to obtain the final estimated value. The final results and computational complexity of each algorithm are given in Table 6, where *n* is the number of samples and *d* is the dimension of the data. It should be noted that in addition to the accuracy, we also give the specificity and sensitivity, which are critical to judge the performance of user experience estimation. These two items are calculated as follows:

(19) $$\begin{array}{c} Sensitivity=\frac{TP}{TP+FN} \end{array}$$

(20) $$\begin{array}{c} Specificity=\frac{FP}{FP+TN} \end{array}$$

in which, TP (true positive) means the KQI was correctly estimated as normal; FP (false positive) means the KQI was incorrectly estimated as normal; TN (true negative) represents that the KQI was correctly estimated as abnormal; FN (false negative) represents that the KQI was incorrectly estimated as abnormal.

In this paper, sensitivity means the proportion of correctly estimated KQIs in KQI normal samples, while specificity is the estimation accuracy of abnormal KQIs. It can be seen that the proposed algorithm and SVM with the Gaussian kernel had the best performance, but the complexity of the proposed algorithm was much less than that of SVM. This is also very important for regional network optimization, because the amount of wireless data in a region is huge, and it is particularly important to select algorithms with low complexity and high effectiveness.

### 4.3. Cell-Level Multi-Service Perception

**Figure 9:**
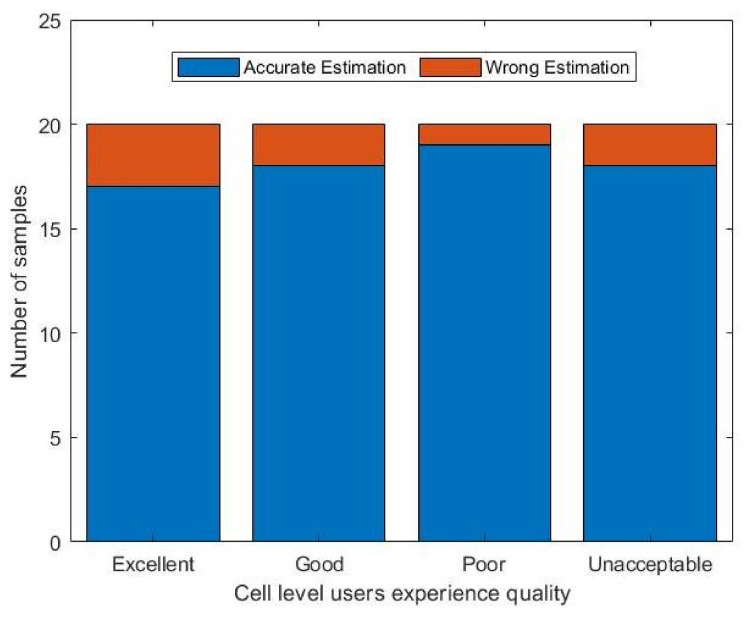
Statistical results for cell-level service perception evaluation.

Cell-level wireless network perception is an overall evaluation index of users for three service types in a cell. The evaluation criteria are shown in Table 4. There are four levels of perception, namely excellent, good, poor, and unacceptable. In the verification process, we selected 80 groups of data, and each level contained 20 groups of data. For the estimation, we combined the voting mechanism with the two-step-optimized AKNB to obtain the estimated KQI for the three services, then the KQIs for three services were summarized into cell-level wireless network perception capability. The statistical results are shown in Figure 9 and Table 7. The proposed method can well estimate the perception capability of cell-level wireless networks.

**Figure 10:**
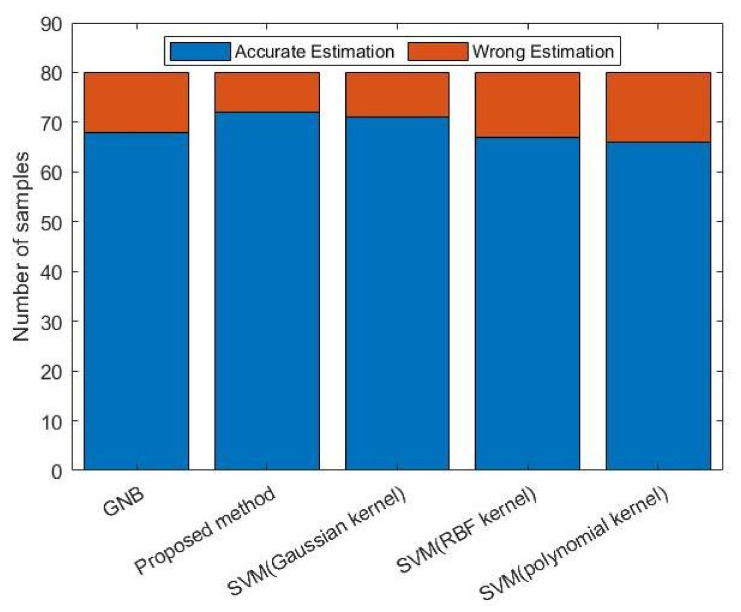
Cell-level service perception evaluation based on different methods.

At the same time, commonly used algorithms in the research of user experience were also selected to evaluate the perception ability of cell-level wireless networks, and the application performance of various algorithms in such scenarios were verified. As the verification in previous step, we used 80 sets of data for the different algorithms. The difference was that we only counted the number of correctly estimated and incorrectly estimated samples, not for different levels of user experience. In our proposed scheme, adaptive kernel density estimation and naive Bayes were used to estimate users’ experience for each type of KPI, and the final user experience was obtained by the voting mechanism; for other algorithms, all KPI data were used to estimate the user experience. These different algorithms were used to estimate the user experience of the three services, respectively, and the estimation results of the three services were summarized as the cell-level wireless network perception capability. Figure 10 shows the results. The proposed algorithm and the SVM based on the Gaussian kernel function had better performance. The algorithms that performed well in user experience prediction can be used to evaluate the perception ability of cell-level wireless networks.

This evaluation standard can well present the cell-level service quality for network provider, and the weight value can be used to summarize the estimated values of three services according to the specific requirements of the network provider for different services in the region. This method can be used to provide guidance and suggestions for network optimization.

## 5. Discussion

We estimated the KQIs for different types of KPIs, respectively, and determined the final estimation result by combining naive Bayes based on two-step optimization in KDE and a voting mechanism. After verification in three aspects, the results showed that compared with traditional KQI estimation algorithms, the accuracy of the algorithm was guaranteed with a low complexity. At the same time, this method combines user prediction with network fault diagnosis, which has not been realized in all previous studies on user experience estimation. The application of the proposed method can find out the KPI that often leads to the decline of user experience in a period of time according to the statistical results, so that the network can be adjusted for such KPIs. Based on the estimation of the user experience, we introduced a wireless network perception capability to evaluate cell-level user experience for multiple services. To the best of the author’s knowledge, this is the first time that multi-service user experience estimation at the cell level has been proposed. The final results showed that the proposed framework can accurately estimate and evaluate the network quality of the cell from the perspective of user experience.

However, some limitations should be noted. In this paper, our data came from one base station due to data collection limitations. The general applicability of the verified method needs to be improved. In the future, we will collect and process city-level data to enhance the generalization ability of the algorithm.

## 6. Conclusions

Recently, in mainstream research on KQI estimation for wireless networks, discretization of KPIs is always adopted, which will lead to some error. In this method, KPI data are regarded as continuous, and prior probability is calculated by the probability density function. For KPI data in wireless networks, the distribution rules of each kind of data are different. For each kind of KPI data, we used the two-step optimized kernel density estimation method to find the best probability distribution through the fitting degree, and this probability density was used by the naive Bayesian classifier to further improve the accuracy. The voting mechanism of KQI estimation was adopted, and the result can feed back to the network optimization personnel which kind of KPI caused the fault, that is which kind of KPI was voted as abnormal. According to the long-term statistical results, we can know which kind of KPI is always problematic. This can make network optimization work more efficiently. The proposed algorithm has low complexity while ensuring accuracy. Finally, we summarized the KQI estimation results of three main services (instant messaging, web page browsing, and video), and the cell-level wireless network perception capability was obtained. The results showed that the proposed method can accurately evaluate the multi-service user perception of a cell.

In the research of user experience estimation, some studies are based on the user’s subjective experience [15,16,20,23]. However, for actual network management, it is not realistic to obtain the subjective experience of each user. Therefore, the objective part of user experience is the KQI, which is increasingly used for research on user experience estimation [21,22,24,25]. The prediction technology of the KPIs for wireless network is becoming mature, but the prediction value of the KPIs cannot well reflect the actual user experience; meanwhile, the KQI data are often unpredictable. Among them, most research is based on discretized KQI data [21]. The effectiveness of the SVM [24,25,26] and Bayesian [13] classifiers has been verified. By comparing the proposed algorithm with these advanced methods, the results showed that the proposed algorithm can better deal with user experience estimation. At the same time, it should be noted that research on user experience estimation and wireless network fault diagnosis is usually in two different research directions. We combined them together and obtained good results in both aspects. The method proposed in this paper can also make the research of wireless network data prediction more practical.

The work of network management and optimization has gradually shifted from manual to network self-management and self-optimization. The proposed method can be well used in network automation. It not only saves network operation cost, but also ensures optimized performance. Our future work will continue to focus on user experience estimation in cellular network. A general model of user subjective experience is the focus of our next research. We hope that the combination for the estimation of the subjective part and the objective part can make more accurate estimation of user experience and more accurate positioning of network faults.

## Figures and Tables

**Table 1:** KPI classification.

KPIs Types	KPIs
*Accessibility*	RRC_establish, E-RAB_establish
*Mobility*	Intra/inter_frequency_handover, Intra/inter_eNB_handover
*Network Quality*	Uplink_MAC_block_error, Downlink_MAC_block_error
*Capacity*	Uplink/Downlink PRB, PUSCH, PDSCH
*Potential Resource*	Average user, Max user, Uplink/Downlink traffic

**Table 2:** KQI classification.

KQIs Types	KQI
*Web service*	Response_success_rate
*IM (Instant messaging) service*	Receiving_success_rate
*Video service*	Playback_success_rate

**Table 3:** Troubleshooting guide.

KPIs Types	Cause of Failure
*Accessibility*	Interference and congestion wireless environment
*Mobility*	Handover parameter configuration and base station identification configuration
*Network Quality*	Hardware failure and strong interference
*Capacity*	Base station failure and weak coverage
*Potential Resource*	Capacity expansion and resource allocation for different services

**Table 4:** Cellular network perception capability.

Cell Level Users Experience Quality	IM	Video	Web Browsing
*Unacceptable*	Abnormal	Abnormal	Abnormal
	Abnormal	Abnormal	Normal
*Poor*	Normal	Abnormal	Abnormal
	Abnormal	Normal	Abnormal
	Abnormal	Normal	Normal
*Good*	Normal	Abnormal	Normal
	Normal	Normal	Abnormal
*Excellent*	Normal	Normal	Normal

**Table 5:** Algorithm comparison for three service KQIs’ estimation.

KQI Types	KPI Types	AKNB	GNB	KNN
	*Accessibility*	0.7845	0.744	0.6528
	*Mobility*	0.8193	0.7957	0.6436
*Video*	*Network Quality*	0.8254	0.8088	0.7822
	*Capacity*	0.8091	0.8095	0.8614
	*Potential Resource*	0.8079	0.8053	0.8119
	*Accessibility*	0.7989	0.7475	0.7873
	*Mobility*	0.7847	0.7254	0.7019
*IM*	*Network Quality*	0.8413	0.8413	0.8515
	*Capacity*	0.8543	0.6782	0.7183
	*Potential Resource*	0.7554	0.6881	0.8465
	*Accessibility*	0.8132	0.7843	0.7962
	*Mobility*	0.7925	0.7854	0.8114
*Web*	*Network Quality*	0.8819	0.861	0.8713
	*Capacity*	0.831	0.8517	0.7908
	*Potential Resource*	0.9009	0.8502	0.8631

**Table 6:** Comparison results of various algorithms.

Video Service	Accuracy	Sensitivity	Specificity	Complexity
KNN	0.7567	0.7526	0.7977	O (nd)
SVM polynomial	0.791	0.7724	0.8352	O (dn${}^{3}$)
SVM RBF	0.7967	0.798	0.7043	O (dn${}^{3}$)
SVM Gaussian	0.8106	0.8212	0.8178	O (dn${}^{3}$)
Proposed method	0.8073	0.8149	0.8237	O (nd)

**Table 7:** Statistical results for cell-level service perception evaluation.

Excellent		Good		Poor		Unacceptable	
*Accurate*	*Wrong*	*Accurate*	*Wrong*	*Accurate*	*Wrong*	*Accurate*	*Wrong*
17	3	18	2	19	1	18	2

## Data Availability

No data available.

## Abbreviations

The following abbreviations are used in this manuscript:

KPI	Key performance indicators
KQI	Key quality indicators
QoS	Quality of service
QoE	Quality of experience
KDE	Kernel density estimation
GNB	Gaussian naive Bayes
AKNB	Adaptive kernel-density-estimation-based naive Bayes
E-RAB	Evolved radio access bearer
RRC	Radio resource control
UE	Users
eNB	Evolved Node B
